# Estimating the Emotional Information in Japanese Songs Using Search Engines

**DOI:** 10.3390/s22051800

**Published:** 2022-02-24

**Authors:** Jin Akaishi, Masaki Sakata, Jouichiro Yoshinaga, Mitsutaka Nakano, Kazuhiro Koshi, Kimiyasu Kiyota

**Affiliations:** 1 Department of Human-Oriented Information Systems Engineering, National Institute of Technology, Kumamoto College, 2659-2 Suya, Koshi 861-1102, Kumamoto, Japan; nakano@kumamoto-nct.ac.jp (M.N.); kkoshi@kumamoto-nct.ac.jp (K.K.); kkiyota@kumamoto-nct.ac.jp (K.K.); 2 School of Engineering, Hokkaido University, Kita 13, Nishi 8, Kita-ku, Sapporo 060-8628, Hokkaido, Japan; sakata.masaki.e9@elms.hokudai.ac.jp; 3 Nisshin Electronics Service Co., Ltd., Tokyo Skytree East Tower F15, 1-1-2, Oshiage, Sumida-ku, Tokyo 131-0045, Japan; hi16yoshinaga@g.kumamoto-nct.ac.jp

**Keywords:** emotion tagging, mood management, Japanese song lyrics, search engine

## Abstract

Several studies have shown that music can reduce unpleasant emotions. Based on the results of this research, several systems have been proposed to suggest songs that match the emotions of the audience. As a part of the system, we aim to develop a method that can infer the emotional value of a song from its Japanese lyrics with higher accuracy, by applying the technology of inferring the emotions expressed in sentences. In addition to matching with a basic emotion dictionary, we use a Web search engine to evaluate the sentiment of words that are not included in the dictionary. As a further improvement, as a pre-processing of the input to the system, the system corrects the omissions of the following verbs or particles and inverted sentences, which are frequently used in Japanese lyrics, into normal sentences. We quantitatively evaluate the degree to which these processes improve the emotion estimation system. The results show that the preprocessing could improve the accuracy by about 4%. Japanese lyrics contain many informal sentences such as inversions. We pre-processed these sentences into formal sentences and investigated the effect of the pre-processing on the emotional inference of the lyrics. The results show that the preprocessing may improve the accuracy of emotion estimation.

## 1. Introduction

Recent studies have shown that music and songs have positive effects on a person’s psychological state and physical health, such as improving concentration and reducing fatigue and stress [1,2,3]. It is becoming clear that negative emotions can be alleviated by listening to music and songs that remind people of a particular emotion. Matsumoto found that when a subject has very sad emotions and listens to sad music, the feeling of sadness decreases [4]. Similarly, a study by Sharman and Dingle revealed that listening to intense music when a subject is feeling angry can alleviate the anger [5]. Based on these research results, a system that proposes songs according to the mood of the listener has been proposed [6]. However, it has also been reported that offensive songs can have a negative impact on the audience [7,8]. In these systems, it is important to be able to avoid songs that may have a negative impact and suggest songs that have a positive impact. The provision of songs to people with serious mental conditions needs to be carefully proposed through the use of a white list to avoid negative effects.

One of the important functions in the song proposal system is to automatically estimate the emotions evoked by the song with higher accuracy. A system that automatically classifies the emotions in a song is indispensable for processing a certain request from a huge number of songs. There have been many studies on methods for inferring emotions in songs [9]. Audio and lyrics can be considered as elements that evoke emotions contained in songs [10,11]. Several systems have been developed to estimate the emotion of a song using the lyrics as the target of analysis [12]. In addition to English, systems for estimating emotions have been developed for Chinese and Japanese lyrics [13,14]. To improve the accuracy of sentiment estimation, it is important to make adjustments that correspond to the unique features of each language.

Kaur and Saini pointed out that the accuracy of sentiment estimation decreases in sentences with an informal writing style [15]. It is important to propose a method to deal with this informal writing style to improve the accuracy of emotion estimation. In the case of Japanese lyrics, substantive stop and inversion are the most commonly used informal writing styles. Few studies have quantitatively evaluated the effects of informal writing styles on the accuracy of emotion estimation for Japanese lyrics.

Several methods have been put forward for extracting emotional information. Estimation methods using machine learning and contextual methods have been proposed [16,17]. In contrast, a classic and simple method is the analysis using an emotion dictionary. This method is easier to analyze than more complex methods and has the advantage that it is easier to understand the impact of adding new elements. One conventional method focuses on surface expression and processes emotions using a valence pattern using an emotion dictionary, ML-Ask, which was developed by Ptaszynski et al. [18]. An emotion evaluation system with more complex functions, such as consideration of negative sentences in Japanese, has also been used [18]. Furthermore, a method has been proposed by Shi [19] to search for related words in text using Web mining [19]. This method improves the accuracy of estimating the emotional value contained in the text. By extracting emotional words that co-occur from a large amount of text on the web for an input phrase and performing emotional estimation, even if there is no direct emotional expression in the song phrase, it is possible to infer the emotion.

There have been many studies aimed at extracting emotional information from sources such as news items, conversations, and song lyrics (e.g., Ptaszynski et al. [18]). There have also been many attempts at emotional estimation for lyrics. However, research on estimating the emotions in Japanese lyrics using a Web search engine is limited [20]. The previous study has shown that the accuracy of the estimation tends to be improved by using Web search for the estimation of emotions in Japanese lyrics. However, because punctuation could be omitted, it might also create subtle differences in meaning. In addition, it has been pointed out that the accuracy of sentiment estimation may be improved by revising sentences with substantive stop or ending a sentence with a noun or inversion to normal sentences. Japanese lyrics use many special grammatical features, such as substantive stops and inversions. By focusing on lyrics that contain many of these grammatical structures, we expect to obtain results that focus on the analysis of emotions in Japanese songs, which is different from research on ordinary literature. However, quantitative evaluation of the improvement in accuracy has not yet been conducted.

Quantifying the effect of modifying sentences with informal writing styles, which is peculiarly common in Japanese lyrics, to normal sentences on sentiment estimation using Web search will be useful from the technical point of view of improving the accuracy of sentiment estimation for Japanese sentences. It will also benefit from the perspective of improving the performance of systems that contribute to mental stability through music.

The purpose of this study is as follows. The object of analysis in this study is the lyrics, not the music. As part of the song proposal method, we use a Web search engine to build a system that can classify the emotions that a song evokes based on Japanese lyric information. We then compare the accuracy of this system with that of conventional methods. Next, we introduce a preprocessing method to correct substantive stops and inverted sentences, which are often found in Japanese lyrics, into a formal writing style and evaluate its effectiveness. In the future, it is envisioned that the system will be part of a system that helps people who are casually listening to music on portable music devices to ease their feelings when they feel somewhat naive or angry.

## 2. Materials and Methods

First, we selected recently popular Japanese songs and extracted the lyrics for testing. Next, the lyrics decomposed into morphemes were input into a system that infers the emotions that the lyrics recall to humans, and the results were output.

Seven emotion categories are dealt with in this study: joy, relief, sadness, anger, like, dislike, and excitement. There are 10 types of classifications widely used in Japanese sentiment analysis in the “Emotional Display Dictionary” [21]. However, when Japanese lyrics are presented to subjects, the emotions that the subjects feel from the lyrics are frequently five types: “joy, like, relief, sadness, and excitement.” It has become clear that the five types of emotion, “dislike, fear, anger, shame, and surprise”, rarely appear [22]. Based on the above results, to reduce the processing burden, this study deals with seven categories by adding two emotions that appeared relatively frequently. Among the five classifications with a low frequency of occurrence, two of them, unlike and anger, have a relatively high frequency of occurrence. We decided to handle seven sentiment categories, considering the balance between accuracy and processing load reduction.

The emotion dictionary used in this study was an extension of the standard emotion word dictionary in Japanese, “Emotional Display Dictionary”. In addition to the 1097 words in the emotional expression dictionary, synonyms for emotional words have been added from the “Must-have Synonyms Practical Dictionary” published by Sanseido [23]. The total number of emotional words after the expansion is 1930.

We used the four systems described below as emotion estimation systems.

1. Method A: The emotion is estimated by matching words in the lyrics with the emotion dictionary. This is a classic and simple method that can be used as a basis for comparison with other methods. First, we divide the lyrics into morphemes. The morphemes are then compared with the headwords in the emotion dictionary. If they match, we add a point to the emotion category assigned to the word in the dictionary. The cumulative total of points for each of these emotion categories is interpreted as the emotion expressed by the lyrics.
2. Method B: The ML-Ask system is used for sentiment analysis of lyrics in the method. The system has complex functions, such as consideration of negative sentences in Japanese. This system is publicly available and has been widely used in sentiment analysis of Japanese sentences.
3. Method C: In this method, we used the ML-Ask system and a Web search engine.
4. Method D: In addition to Method C, we added a pre-processing step to modify lyrics with special syntax into normal sentences before using the Web search engine.

Table 1 shows the processes used in these methods. A checkmark indicates that the process was used. Of these four methods, we conducted Methods A through C as Experiment 1, and Method D as Experiment 2.

### 2.1. Experiment 1

First, we selected 50 recently popular Japanese songs and extracted the lyrics for testing. Then, the lyrics of the 50 songs were shown to 10 subjects (eight males and two females, in their teens to 40 s, students, mean age: 22.4 years) on a website. Subjects were asked to choose from seven different emotions (joy, relief, sadness, anger, like, dislike, and excitement) that were evoked when they read the lyrics, and to vote by clicking on the corresponding emotion button on the homepage (multiple responses were allowed). The top three emotions were used as the majority of data for the human evaluation of the emotions.

#### 2.1.1. System for Classifying Lyrics Emotions

**Figure 1:**
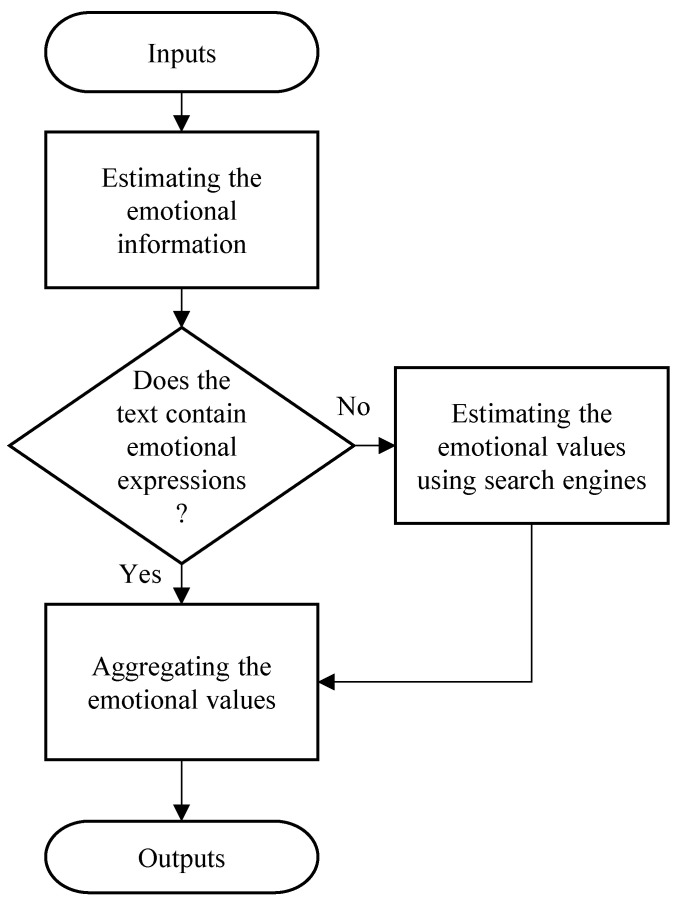
The flowchart of the system for classifying lyrics emotions.

We describe in detail a method (Method C) that uses a Web search engine to infer the emotions evoked by the lyrics we propose this time. An outline of the system is shown in Figure 1. In the proposed method, emotions are first estimated using ML-Ask. Here, we use a method for inferring emotions from sentence-ending expressions and emotional expressions contained in the sentence itself using a superficial expression of the sentence. If the emotion expression cannot be extracted, an emotion estimation using a search engine, “emotion estimation by causal relation on the Web”, is performed. Here, we used a method of acquiring sentences that have a causal relationship with sentences from the Web and recognizing emotions. We processed the lyrics in the following steps:

1 Divide the lyrics into morphemes with MeCab [24].
2 Analyze the lyrics divided into morphemes.
  2.1 Outputs the emotion value for the matching classification when the morpheme matches the emotional word in the dictionary of the system.
  2.2 If there is no emotional word in the sentence, analyze the sentence that holds the causal relationship that exists on the Web.
    2.2.1 Divide the sentences from the Web into morphemes with MeCab.
    2.2.2 Comparison with an emotion dictionary, and if they match, output the emotion values of each emotional classification.
3 Aggregate the output emotional value

The flow of this process is shown in the flowchart in Figure 1.

The following is an example of the process. For example, if a lyric contains an emotional word such as “happy”, the word “joy” is judged. If the lyrics do not contain emotion words, such as “I played baseball”, the system uses a search engine to retrieve sentences on the Web that are related to the lyrics. For example, if the sentence retrieved is “I had fun playing baseball”, the co-occurrence of the emotion word “fun” is judged as “joy”.

#### 2.1.2. Emotion Estimation Using Search Engine

This section describes the detailed procedure for emotion estimation using a search engine. When a lyric phrase does not contain a word with an emotion in the dictionary, the phrase in the lyric is extracted and a conjunction is added to the end of the sentence to create a search query. The search query is input and the resulting short sentences are saved. The search query is created based on the rules used by Shi [19], and some modifications are made according to the emotion estimation of the lyrics. The following is a description of these modifications:

- Creating phrases from 3-grams
  ➢ If the following conditions are not met, lengthen it to 4-grams, 5-grams, etc.
- The beginning of the phrase is not a particle or conjunction
  ➢ Do not make phrases from particles or conjunctions
- The end of the phrase is a verb or adjective
- Ignoring stop words
- Changing the end of a sentence to a non-past terminal form
  ➢ Making the phrase grammatically correct when a conjunction is added

Next, to make it easier for emotional words to co-occur, the Japanese conjunctions “*-te, -to, -node*” are added to the end of the created phrase. A search query is a combination of these three conjunctions with a phrase. Shi revealed that by including some adjunct words in the search query, more search results including emotional words can be obtained [19]. Based on the results, we adopted the above three conjunctions, which had a high probability of acquiring emotional words. The reason for limiting the number of conjunctions to three is to limit the amount of searching.

We obtained snippets from a search engine using a query with the conjunctions described above. In this study, we used Microsoft Bing as the search engine. Forty snippets obtained for each query were saved as text data. In other words, one phrase saves 120 snippets. From the acquired snippets, the sentences following the input phrase were saved for use in emotional estimation. However, if the sentence following the phrase contains a conjunction such as “but”, the sentence was not saved. Table 2 shows an example of the above process.

#### 2.1.3. Analysis of Text Data

Morphological analysis was performed on the saved text data, and matching processing was carried out using a dictionary that is an extended “Emotional Expression Dictionary”. Similar to Shi’s research, the number of words that matched the headwords of each emotion category was used as the score for each emotion category. Each emotion category is sorted in descending order of score, and output by the system as the emotion that the song has. Multiple sentiment classifications can be output from the system. To simplify the processing, we decided not to output the emotion categories with low scores according to the following rules. Emotional categories that did not satisfy the following two conditions were ignored and output was set to zero. (1) The score of the emotion category must be in the top 60% of all scores. (2) The score of the emotion category must account for 10% or more of the total score. For example, in the case of “Sad 51%, Joy 9%, and the other five emotions are 8% each”, Joy is in the top 60%, but only sad emotions are output.

#### 2.1.4. Aggregation of Emotional Value

The evaluations for each emotion category obtained by the emotion evaluation by ML-Ask and the emotion evaluation by the causal relationship on the Web are aggregated. The evaluation using ML-Ask, which is processed in the first stage, performs advanced processing, such as directly analyzing the lyrics and considering negative sentences. Therefore, it is considered to be more accurate than emotion estimation using a search engine. Therefore, in order to improve the accuracy, the output of the emotion category when emotional expression by ML-Ask is included is weighted. Five types of weights (1–5 times), were tried, and three times, which had a high F-score overall, were adopted. In other words, in this system, the evaluation by ML-Ask was three times heavier than the evaluation using the Web.

### 2.2. Experiment 2

The key point of the emotion prediction using Method D in Experiment 2 is the pre-processing before inputting the lyrics into the system used in Method C. In the pre-processing, the lyrics were divided into individual sentences to determine whether informal writing styles were used or not. If informal writing styles were used, the sentence was modified to a formal writing style by the process described below. We then input phrases into the system as in Method C of Experiment 1 and evaluated the accuracy of the emotion guessing. We describe the details of the preprocessing in Experiment 2 below.

- Substantive stop or ending a sentence with a noun: In Japanese, sentences usually end with a verb, and the substantive stop is a special construction that means to end a sentence with a noun or pronoun. This construction is often used in lyrics. When a sentence ends with a noun or a pronoun, and there is a verb in the sentence, the noun is moved to the front of the verb in the sentence, and the particle is added to the end of the sentence to convert it into a normal sentence.
- Inversion: In an inversion, the subject “noun + particle” is placed at the end of the sentence. When a sentence ends with a “noun + particle”, the “noun + particle” is moved to the beginning of the sentence.
- Omission of verbs: If the lyrics end with a noun and there is no verb in the sentence, we added the omitted verb and the related word.
- Processing sentences enclosed in parentheses or quotation marks: In previous studies, many codes were excluded from the process as stop words. In this study, however, sentences or words in parentheses are treated as separate sentences.

In Experiment 2, 30 songs different from those in Experiment 1 were selected, and 15 people (11 males, 4 females; all students are in their twenties; mean age 20.0 years old) voted for the seven emotions (multiple responses were possible), and the top three were evaluated as the majority answer. Since the data were different from that of Experiment 1, emotion estimation was also conducted using Method A, which evaluates only the emotion dictionary, as a standard for comparison.

### 2.3. Evaluation by F-Score

We use the F-score as a measure of the prediction accuracy of the system. The process of obtaining the F-score is described below.

The seven emotion categories used in this study are denoted as set A:

(1) A :={joy,relief,sadness,anger,like,dislike,excitement}.

Using the lyrics of a song as input, the system outputs the probability of each emotion category. Next, the system eliminates the emotion categories with low probability according to the conditions described in Section 2.1.3. Finally, the system selects up to three emotional categories from among the ones with the highest probability, and outputs them as the final output. Let Es ⊂ A be the set of emotion categories predicted by the system, where Es is a subset of A. Similarly, let Eh ⊂ A be the set of emotion categories evaluated by a human after reading the lyrics of a song, where Eh is a subset of A.

The sets TP, FP, FN, and TN required for the calculation of the F-score can be obtained by the logical operation shown in the following equations:

(2) TP=Eh∧Es, FP=¬Eh∧Es, FN=Eh∧¬Es, TN=¬Eh∧¬Es.

Using the sets TP, FP, FN, and TN, Precision and Recall are obtained:

(3) $$\mathrm{Precision}=\frac{n\left(\mathrm{TP}\right)}{n\left(\mathrm{TP}\right)+n\left(\mathrm{FP}\right)},$$

(4) $$\mathrm{Recall}=\frac{n\left(\mathrm{TP}\right)}{n\left(\mathrm{TP}\right)+n\left(\mathrm{FN}\right)}.$$

Here, n(X) is a function to find the number of elements in the set X. Using this Precision and Recall, we calculated the F-score:

(5) $$\mathrm{Fscore}=\frac{2\mathrm{Precision}\cdot \mathrm{Recall}}{\mathrm{Precision}+\mathrm{Recall}}.$$

## 3. Results

The accuracy was compared by calculating the F-score (F1) for the emotion estimation results for the systems. The results of Experiment 1 are shown in Table 3.

The results of Experiment 2 are shown in Table 4. In the results shown in Table 4, the F-score of Method A is 58% and that of Method D is 67%. Note that the results for Method A, here, evaluate a different dataset than in Experiment 1.

## 4. Discussion

Comparing the F-values for Method C and Method B, Method C is 13% higher than Method B. Therefore, emotional estimation using causal relationships on the Web is effective. Comparing Method C and Method A, the recall rate improved by 8%, and the F-score improved by 7%. Therefore, the accuracy improved slightly.

The reason why Method A has a higher F score than Method B, even though the procedure is simpler, is considered to be because the emotion dictionary is larger than that for Method B. The dictionary used in Method A has 1930 emotion words, and Method B has 907 emotion words. The emotion dictionary used in Method C that evaluates sentences from the Web is the same as the dictionary in Method A.

There is a study by Ptaszynski using a Web search for emotion estimation, which was the basis of this study [18]. In their study, the F-score was 54% to 53% when the emotions of conversational sentences were estimated by a similar method. It is difficult to make a simple comparison with the results of the present study because the analysis target, the number of emotion categories, and the types of search engines are different. However, the emotion estimation of the lyrics in this study is about 7% better than the case for conversational sentences. This indicates that the use of a Web search engine may be more suitable than the analysis of conversational sentences in estimating emotions in lyrics.

The introduction of Method D improved the F-score by 7 compared to Method C. However, the datasets of Experiment 1 and Experiment 2 are different. Based on the difference between the results of Method A in each experiment, it can be inferred that the dataset in Experiment 2 tends to be easier to guess the emotion by 4 points. Even after subtracting these four points, the change to normal sentences tended to be effective.

There are several reasons why this change to normal text was effective, including the following. Table 5 shows an example of lyrics expressed using three types of methods, i.e., a normal sentence, a word stop, and an inversion method, and phrases generated from the sentence. Since the three original sentences in Table 5 do not contain emotion words, emotion inference based on causal relationships on the Web is applied. Therefore, phrase creation for the search query is performed. The phrases generated based on the phrase creation rules are shown on the right-hand side of Table 5.

As can be seen in the generated phrases in Table 5, the word “meteor shower” is omitted from the phrases generated based on the sentences using the word-stopping and inversion methods. This is because the phrase creation rule “the end of the phrase is a verb or an adjective” is applied. By eliminating the word “meteor shower” in the search query, the resulting snippet becomes more abstract and the estimated emotion value can be different from that in the original lyrics. Therefore, the accuracy of the sentiment classification was improved by judging the sentences with substantive stop and inversion in advance and processing them back to plain text.

It was shown that Method C and D in this study improve the accuracy of emotion evaluation. However, this improvement in accuracy is limited when the cost of the network and the computational cost of using a search engine are taken into account.

This study was limited in several aspects due to the limitations of the research resources. The following is a discussion of these limitations.

The first limitation is the number of samples obtained as the target data. The sample size of this study is 10, and the age range is limited, so there is a limit to the generalization of the target data with the system. In the future, a more accurate comparison with the target data will be required by acquiring more data.

Next, there is a linguistic limitation. In this study, we used Japanese lyrics, so our findings are limited to the scope of Japanese.

The third limitation is the technical limitation. In the improved method of sentiment estimation using Web search engines used in this study, the search results are not stable because the search results of the engines are updated daily. In addition, it is necessary to balance the cost of using the network and creating search queries with the benefit of improving the accuracy obtained.

Furthermore, in the estimation of emotions evoked by songs, combining the analysis of lyrics and music is expected to achieve higher accuracy. Music is also an important factor in evoking emotions. The emotions evoked by lyrics can be diversified depending on various factors such as the background of the listener. In addition, music may evoke emotions opposite to those of lyrics [8]. Therefore, the accuracy of emotion estimation may be limited by analyzing only the lyrics. Due to the limitations of our expertise and research resources, we limited the scope of our study in this paper to Japanese lyrics. In the future, it is expected that the findings obtained in this study will contribute to the improvement of more accurate emotion analysis techniques by combining them with music analysis.

## 5. Conclusions

In this study, we constructed a system that can classify the emotions that a song evokes from Japanese lyric information using a Web search engine as part of a song proposal system that suits the mood of the listener. We also performed a quantitative evaluation using a method that combines emotion estimation using a superficial expression of sentences and emotion estimation using a search engine. The method using a search engine improved the accuracy by 4% compared to the method using a conventional dictionary.

In addition, we attempted to improve the accuracy of sentiment inference by introducing preprocessing techniques such as substantive stops and inversions to normal sentences. The results show that the accuracy could be improved by about 4%. However, it is difficult to make an exact comparison because the datasets used in the experiments are different.

As a future development, we will implement a system that can suggest songs suitable for the user’s emotions using our results. If future research focuses on improving song recommendation logic, it should reflect findings related to music psychology and music therapy, taking into account that people with serious mental health conditions may use the system. There is a method to measure the effect of a certain function called ablation experiment, which is often used in deep learning research. In this method, the effect of a function is measured by pausing the function and checking the effect of the paused function. To clarify the effect of Web search, it is possible to conduct ablation experiments.

## Figures and Tables

**Table 1:** Processing techniques used in each method.

	Emotion Dictionary	ML-Ask	Web Search Engine	Pre-Processing
Method A	✓			
Method B	✓	✓		
Method C	✓	✓	✓	
Method D	✓	✓	✓	✓

**Table 2:** Example of inputting Japanese lyrics to saving the sentence to be analyzed when estimating emotions by Web search.

	*Romanized Transcription*/English Translation
Input sentence	*Harukaze ga fuite ita.*/Spring breeze was blowing.
Phrase	*Harukaze ga fuku*/Spring breeze blows
Search query	*Harukaze ga fukuto*/When the spring breeze blows
Snippet	*Hito wa harukazegafuku to haru no torai wo shiri, atatakaku naru to omotte yorokobu*/People are pleased to know the arrival of spring when the spring breeze blows and think that it will be warm.
Saved sentence	*Haru no torai wo shiri, atatakaku naru to omotte yorokobu*/(People are) happy to know the arrival of spring and think it will be warm

**Table 3:** Results when the majority answer data are in the top three estimated emotions by Web search.

	Precision [%]	Recall [%]	F-Score [%]
Method A	63	50	54
Method B	66	40	48
Method C	67	58	61

**Table 4:** Results when the majority answer data are in the top three estimated emotions by Web search.

	Precision [%]	Recall [%]	F-Score [%]
Method A	65	55	58
Method D	72	66	67

**Table 5:** Effect of inversion and ending a sentence with a noun in generated phrases.

	Original Sentence	Generated Phrases
	*Romanized Transcription*/English Translation	
Normal sentence	*Ryuseigun ga yozora ni kiete itta.*/The meteor shower disappeared into the night sky.	*Ryuseigun ga yozora ni kieru.*/Meteor shower disappears in the night sky. *Yozora ni kieru.*/Disappear in the night sky
Ending a sentence with a noun	*Yozora ni kiete itta ryuseigun*/A meteor shower that disappeared in the night sky	*Yozora ni kieru.*/Disappear in the night sky
Inverted sentence	*Yozora ni kiete itta, ryuseigun ga.*/The meteor shower that disappeared in the night sky.	*Yozora ni kieru.*/Disappear in the night sky
